# Importance of heterogeneity in *Porhyromonas gingivalis* lipopolysaccharide lipid A in tissue specific inflammatory signalling

**DOI:** 10.1080/20002297.2018.1440128

**Published:** 2018-02-26

**Authors:** Ingar Olsen, Sim K. Singhrao

**Affiliations:** ^a^ Department of Oral Biology, Faculty of Dentistry, University of Oslo, Oslo, Norway; ^b^ Dementia and Neurodegenerative Diseases Research Group, Faculty of Clinical and Biomedical Sciences, School of Dentistry, University of Central Lancashire, Preston, UK

**Keywords:** *P. gingivalis*, lipid A, heterogeneity, penta-acylated form, tetra-acylated form, innate immunity subversion

## Abstract

Lipopolysaccharide (LPS) of *Porphyromonas gingivalis* exists in at least two known forms, O-LPS and A-LPS. A-LPS shows heterogeneity in which two isoforms designated LPS_1_,_435/1_,_449_ and LPS_1_,_690_ appear responsible for tissue-specific immune signalling pathways activation and increased virulence. The modification of lipid A to tetra-acylated_1_,_435/1_,_449_ and/or penta-acylated_1_,_690_ fatty acids indicates poor growth conditions and bioavailability of hemin. Hemin protects *P. gingivalis* from serum resistance and the lipid A serves as a site for its binding. The LPS_1_,_435/1_,_449_ and LPS_1_,_690_ isoforms can produce opposite effects on the human Toll-like receptors (TLR) TLR2 and TLR4 activation. This enables *P. gingivalis* to select the conditions for its entry, survival, and that of its co-habiting species in the host, orchestrating its virulence to control innate immune pathway activation and biofilm dysbiosis. This review describes a number of effects that LPS_1_,_435/1_,_449_ and LPS_1_,_690_ can exert on the host tissues such as deregulation of the innate immune system, subversion of host cell autophagy, regulation of outer membrane vesicle production, and adverse effects on pregnancy outcome. The ability to change its LPS_1_,_435/1_,_449_ and/or LPS_1_,_690_ composition may enable *P. gingivalis* to paralyze local pro-inflammatory cytokine production, thereby gaining access to its primary location in periodontal tissue.

## Introduction

**Figure 1.**
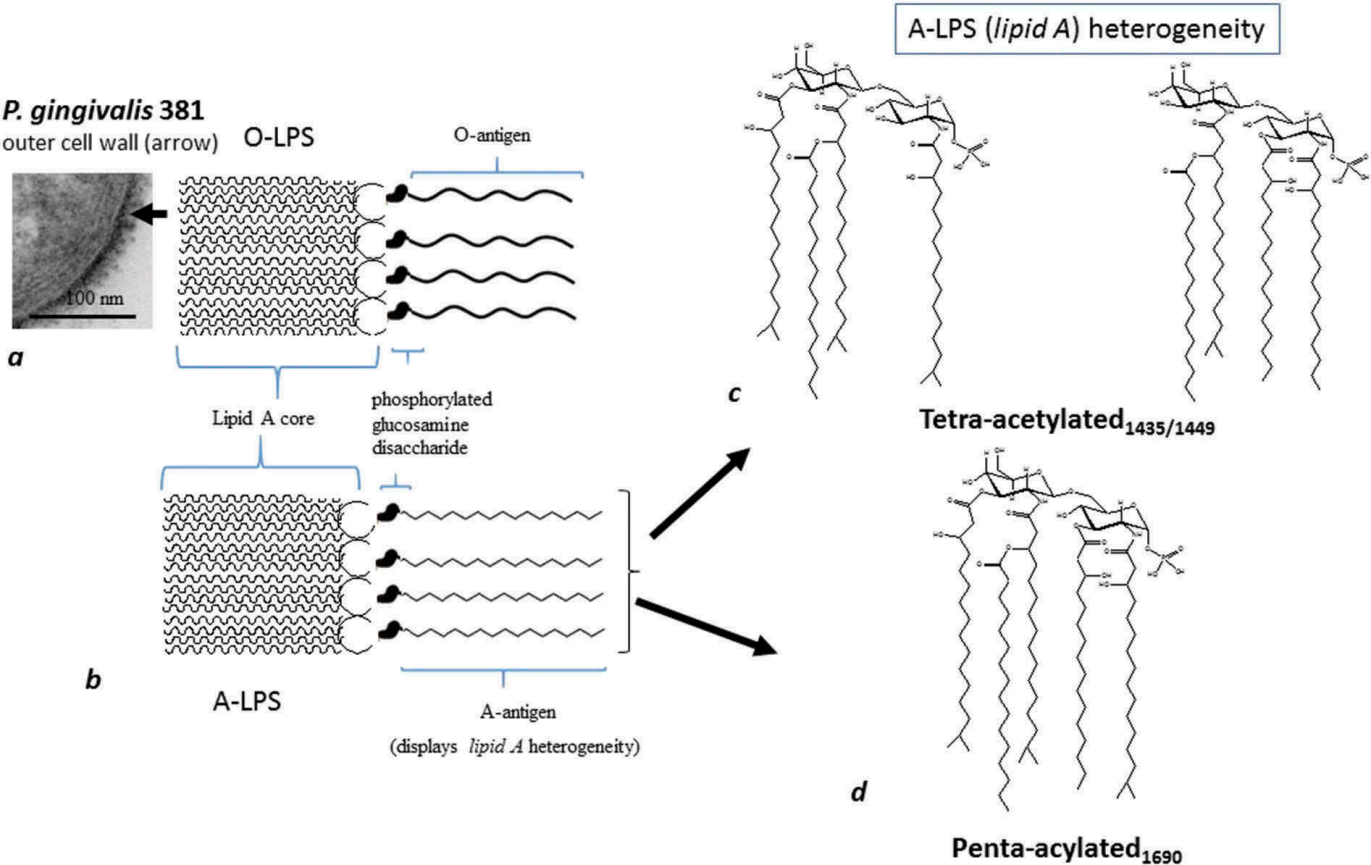
Schematically shows two main forms of LPS (O-LPS and A-LPS) and their position on the outer cell wall (arrow). (a) Section of *P. gingivalis* (FDC 381) demonstrated by transmission electron microscopy. Thick arrow points to lipid A with fatty acid esters attached to phosphorylated glucosamine disaccharides and the *O*-antigen. The latter is O-LPS (with *O*-antigen tetrasaccharide repeating units); (b) A-LPS (with surface anionic polysaccharide [APS] repeating units). There are two lipid As in A-LPS as shown in (c) and (d). (c) A tetra-acylated form with two different molecular weights, hence two structures. (d) the penta-acylated form.

*Porphyromonas gingivalis* is considered to be a keystone Gram-negative, anaerobic intracellular pathogen in adult periodontitis [1]. It has a plethora of virulence factors [2] of which lipopolysaccharide (LPS), gingipains, fimbriae, hemagglutinins, and outer membrane vesicles (OMVs) are of major importance. LPS is located in the outer membrane of Gram-negative bacteria and is a potent stimulator of host’s innate immune signal transduction pathways in a tissue/cell-specific manner [3]. This is seen in bone, epithelial cell barrier breakdown, and keratinocytes [4,5]. Historically, LPS has been thought to consist of three variable and conserved regions [6,7]. These include lipid A (common to all forms of LPS from Gram-negative bacteria), which can consist of fatty acid esters; these fatty acid esters in turn are either attached to phosphorylated glucosamine disaccharides or 3-OH isobranched C17:0 and other fatty acids with amide-links to both saccharide units in lipid A: a conserved core oligosaccharide that links lipid A to the *O*-antigen; and a highly variable *O*-polysaccharide or *O*-antigen [8] (Figure 1).

Of the LPS macromolecule, lipid A is responsible for the endotoxic activities, and its recognition by host cells leads to differential immuno-inflammatory responses [4,7–9]. From LPS, it is the heterogeneity within the A-LPS which represents the major virulence factors that promote inflammation and bone loss. Hence in immunological terms, the A-LPS heterogeneous forms represent ‘pathogen associated molecular patterns’ (PAMPs) and have been extensively studied for their role in the pathogenesis of periodontitis (for a review, see Nichols et al. [10]), however not without controversy. Whereas some studies reported that *P. gingivalis* LPS stimulates secretion of pro-inflammatory cytokines [11], others found contradictory results with regard to cytokine release [5,12]. Besides, LPS acting as an agonist for Toll-like receptor (TLR) TLR2 or as an antagonist and/or agonist for TLR4 activation [13–16] added to further contradiction. Although TLR2 activation by *P. gingivalis* LPS is possible, the difference lies in the form of LPS presented to the host. For example, the protein-free LPS is unable to activate TLR2, whereas the bound LPS on the live bacterium can mediate TLR2 activation via a novel class of lipoprotein lipase-sensitive molecules highlighting the importance of active infection [17]. In essence, and according to Lasica et al. [18], the tightly associated or covalently attached protein to LPS on live *P. gingivalis* accounts for the TLR2 activation. In terms of bone loss, *in vitro* and *in vivo* experiments have provided conflicting data. For example, when *P. gingivalis* is co-cultured with bone cells *in vitro*, the effect on bone resorbing cells appears to be mediated through engagement of both TLR2 and TLR4 [15,19]. However, similar experiments conducted in experimental animals orally infected with *P. gingivalis* predominantly show bone loss to be mediated by TLR2 [20–22]. If the *in vivo* data are confirmed in human alveolar bone resorption mechanisms, this is another example of *P. gingivalis* LPS lipid A heterogeneity showing differential inflammatory signalling according to tissue specificity.

*P. gingivalis* has two major forms of LPS, O-LPS (Figure 1(a)) [23] and A-LPS (Figure 1(b)) [24–26]. O-LPS is a conventional *O*-antigen polysaccharide found in most bacteria with Gram-negative characteristics, and A-LPS is an anionic polysaccharide (APS) (Figure 1(b)). Both O and A-LPS forms are linked to lipid A. While the *O*-antigen repeating polysaccharide unit of O-LPS contains →3)-α-d-Gal*p*-(1→6)-α-d-Glc*p*-(1→4)-α-l-Rha-(1→3)-β-d-GalNAc*p*-(1→, the polysaccharide repeating unit of A-LPS consists of a phosphorylated branched d-Man-containing oligomer made up of an α1→6-linked d-mannose backbone. To the latter branch of the backbone, α1→2-linked d-Man side chains of varying lengths with one or two residues are attached at position 2 [27].

In addition to the structural differences in *P. gingivalis* A-LPS mentioned above, a variety of different lipid A structures have been reported [28,29]. These lipid A structures, in the literature, are referred to as phosphorylated tetra-acylated and phosphorylated penta-acylated proteins. The tetra-acylated form of A-LPS has a molecular weight of 1,435 and 1,449 Da, and the penta-acylated form of A-LPS has a molecular weight of 1,690 Da [30]. For clarity, the tetra-acylated form of A-LPS has been designated LPS_1_,_435/1_,_449_ (Figure 1(c)) and the penta-acylated form LPS_1_,_690_ (Figure 1(d)) [30]. The role of these variable regions appears to involve signalling pathway activation in various effector cells, organs, and diseases (Table 1). It has to be stressed that the heterogeneity is related to the bioavailability of essential growth nutrients such as hemin [25], phosphate availability [31], and to an extent on temperature [32]. The aim of this review is to assess the importance of the heterogeneity in *P. gingivalis* LPS lipid A.

**Table 1.** Heterogenous tetra- and penta acylated A-LPS isoforms involved in immune signal transduction pathways in various cell types.

A-LPS	Signalling pathway activated	*Genes* (up regulated)	*Genes* (down regulated)	Effector cells/organ/disease	Reference
LPS_1_,_435/1_,_449_ and LPS_1_,_690_	NF-κB	ELK1, HRAS, IL-1β, TLR-4, TLR-5, TLR-9, TNF, TRAF6, and UBE2N	BTK, IL-2, IRAK1, LTA, CD180, MAPK8IP, NFKBIL1, SIGRR, TIRAP, TLR-1, and TLR-7	Human gingival fibroblasts/gingivae/ periodontitis	[4]
LPS_1_,_435/1_,_449_	p38 MAPK and ERK1/2 P’ways		Unaffected *genes*: SAPK/JNK and AKT		[4]
LPS_1_,_690_	NF-κB and p38 MAPK and ERK1/2 P’ways NF-κB and p38 MAPK	GM-CSF, CXCL10, G-CSF, IL-6, IL-8, CCL2 and TLR4 NFKBIA, NFKB1, and IKBKB. MAP2K4 and MAPK8, E-selectin		Human gingival fibroblasts/gingivae/periodontitis Human oral keratinocytes	[4, 5, 50]

## Lipid A phosphatase is required for colonization and commensal overgrowth of bacteria in periodontitis

*P. gingivalis* has the ability to change its A-LPS lipid A phosphate composition (LPS_1_,_435/1_,_449_ or LPS_1_,_690_) in response to differing environmental conditions. It is this property, which makes the bacterium highly adaptable to different inflammatory niches. *P. gingivalis* can produce A-LPS lipid A structures that are agonists (LPS_1_,_690_) or antagonists (LPS_1_,_435/1_,_449_) to activation of TLR4 [25,31,32] because under the influence of *P. gingivalis*, it helps to create an inflammophilic environment for selection of its co-species and control any competition. Despite the fact that phosphatases are important for successful colonization of *P. gingivalis*, Zenobia et al. [33] demonstrated in a rabbit ligature model of experimental periodontitis that exposure of the oral cavity to the mutant strains (with locked lipid A structures) did not increase the microbial load. This suggests that the mutant strains were unable to remodel their lipid A structure, which would otherwise be capable of inducing a favourable co-inhabiting environment. This begs the question: why would *P. gingivalis* change its lipid A phosphate composition? The answer lies in the Zenobia et al. [33] study that detected significant qualitative changes in the microbial composition from the mutant strains, in that all *P. gingivalis* strains and their LPS preparations resulted in the development of periodontitis. Furthermore, this keystone bacterium even in its mutant form changed the lipid A composition by utilizing the phosphatases from its microenvironment. Hence, successful colonization took place whereby the microbial load increased in the rabbit ligature model of periodontitis [31]. These results suggest that not only disruption of host homeostasis is highly plausible but also that an evolving commensal microbial community can become dysbiotic even under the influence of a mutant form of *P. gingivalis* resulting in disease.

## The lipid A structure is modulated by hemin and temperature

Heme [Fe(II)-protoporphyrin IX] and hemin [Fe(III)-protoporhyrin IX-Cl] are important for the growth, survival, and virulence of *P. gingivalis* [34,35]. *P. gingivalis* cannot synthesize the protoporhyrin IX ring and does not have siderophores for alternative bioavailability of hemin [36–39]. Therefore, *P. gingivalis* depends on the host for a supply of heme [40]. The lysis of multiple erythrocytes, in order to feed *P. gingivalis* heme, would lead to decrease in oxygen for the host, which would in turn contribute to ischemia. Therefore, the bacterium gains advantage for ideal growth conditions. However, if the supply of hemin is less than adequate, as tested *in vitro*, for the pathogen, at least, it is proposed that the A-LPS lipid A structure of *P. gingivalis* becomes modified according to the hemin concentration in the growth medium [25]. Different isoforms of A-LPS have been demonstrated in experiments where *P. gingivalis* cultures contained either low or high hemin concentrations (Figure 1(c,d). At high hemin concentration, LPS_1_,_435/1_,_449 (high hemin)_ was synthesized, and it elicited a weak immune cell activation via TLR2. Biochemical analysis of the lipid A region demonstrated a mono-phosphorylated, tetra-acylated structure (Figure 1(c)) [25]. Alternatively, *P. gingivalis* culture from low hemin concentration, LPS_1_,_690 (low hemin)_, was found to be more immunogenic [25]. Biochemical analysis showed that the LPS activity, surprisingly, was of antagonist nature with a mono-phosphorylated penta-acylated lipid A (Figure 1(d)) [25]. It is worth noting that *P. gingivalis* LPS_1_,_435/1_,_439_ and LPS_1_,_690_ mixtures obtained from culture conditions with high hemin content also demonstrated a potent immunogenic response. These observations, taken together, suggest that *P. gingivalis* LPS_1_,_435/1_,_439/1_,_690_, may have tools to paralyze the host’s innate defences and expose tissues to an inflammophilic milieu [41,42]. One advantage of the heterogeneity displayed in *P. gingivalis* A-LPS would be that at least it would provide resistance from septicaemia/endotoxin shock to the host.

Coats et al. [31] found that *P. gingivalis* uses endogenous lipid A 1 activity to produce a unique non-phosphorylated lipid in A-LPS. This immunologically silent lipid A has the capacity to provide a highly effective mechanism, which is used by this bacterium to evade TLR4 sensing and to resist killing by cationic anti-microbial peptides released by the host. As the lipid A 1-phosphatase activity was suppressed by hemin, it has been suggested that hemin-dependent regulation of lipid A 1-dephosphorylation could change the A-LPS lipid A activity from TLR4 evasive to TLR4 suppressive. This may alter the critical interaction between *P. gingivalis*, the local microbial community, and the innate immune system of the host. Hemin acquisition proteins such as gingipain K (Kgp) and/or the heme receptor (HmuR) molecules [25,43] appear to be the sensors of its concentration in the environment, although other proteins for hemin binding or its transport may also exist.

A-LPS of *P. gingivalis* serves as a matrix for the deposition of µ-oxo-bisheme [[Fe(III)PPIX]_2_O] and is accountable for the black pigment seen within these cells. µ-Oxo-bisheme pigmentation is a form of a bacteriocin and a virulence factor of *P. gingivalis* [40]. Both Arg and Lys gingipains are required for the deposition of this characteristic black pigment. Absence of A-LPS in the extracellular surface of *P. gingivalis* diminishes the scaffold/anchoring mechanism that otherwise retains Arg- and Lys-gingipains [40]. This implies that A-LPS serves as a site for deposition/binding of hemin. Since pigmentation depends on the presence of A-LPS and Arg- and Lys-gingipains, these observations suggest a significant role of A-LPS in the eventual virulence of *P. gingivalis*.

Curtis et al. [32] discovered that *P. gingivalis* at normal body temperature mainly produced non-phosphorylated and mono-phosphorylated tetra-acylated lipid A structures. This phenomenon was tested by culturing *P. gingivalis* at higher temperatures (39 or 41°C), so mimicking the conditions at the sites of periodontal inflammation. This resulted in generating increased amounts of both mono-phosphorylated and penta-acylated lipid A. The final nail in the coffin for *P. gingivalis* was the activation of the TLR4 at higher temperatures, which could potentially kill it via β-defensins 2 and 3 activities. If modification of lipid A by the host’s body temperature variation affects the virulence and susceptibility to killing, then this inflammophilic bacterium may have evolved hidden mechanisms to thrive under precisely those conditions *in vivo*.

## LPS heterogeneity clarifies earlier contradictory results related to host innate immune mediators

It has become apparent that the A-LPS of *P. gingivalis* plays an important role in deregulating the innate immune system of the host. This is further achieved by changing the host’s defence signalling mechanisms through its heterogeneous A-LPS lipid A structures [31,44]. For example, Herath et al. [4] observed that the NF-ĸB signalling pathway was noticeably activated in human gingival fibroblasts (HGFs) by LPS_1_,_690_ but not by LPS_1_,_435/1_,_449_. The heterogeneity displayed in the A-LPS also modulated the secretion of a different profile of the pro-inflammatory cytokine expression such as IL-6 and IL-8 in HGFs [45]. Furthermore, *P. gingivalis* LPS_1_,_690_ significantly up regulated the expression of IL-6 and IL-8 mRNA at the gene level in HGFs, whereas LPS_1_,_435/1_,_449_ failed to do so [45]. In human monocytes, the A-LPS induced production of IL-1α, IL-1β, IL-6, and IL-8, although at a lower rate than that induced by lipid A obtained from total A-LPS [26]. In addition, pro-inflammatory genes significantly up regulated by LPS_1_,_690_ (*GM-CSF, CXCL10, G-CSF, IL-6, IL-8*, and *CCL2*) were down regulated by LPS_1_,_435/1_,_449_ [4] (see Table 1). HGFs matrix metalloproteinase (MMP)-3 and its protein were strikingly up regulated by penta-acylated *P. gingivalis* LPS_1_,_690_ and hexa-acylated *Escherichia coli* LPS but not by tetra-acylated *P. gingivalis* LPS_1_,_435/1_,_449_ [30], suggesting plausible crosstalk between LPS signalling from Gram-negative pathogens during mixed infections. Furthermore, human beta-defensins, hBD-1, hBD-2, and hBD-3 mRNAs, were significantly up regulated in human epithelia by *P. gingivalis* LPS_1_,_690_ but down regulated by LPS_1_,_435/1_,_449_ [46]. Differential signalling pathway activation in various cell types [4] helps explain (at least in part) the contradictory results reported by earlier studies [28,29] and demonstrates tissue-specific modes of inflammatory signals initiated essentially by the same immunogen. The advantage to the keystone bacterium would likely be in suppressing, some signalling pathways for adapting to different tissue environments. This may explain the role *P. gingivalis* plays in establishing disparate organ-specific inflammatory pathologies.

In human oral keratinocytes (HOKs), *P. gingivalis* penta-acylated mono-phosphorylated LPS_1_,_690_ has been shown to act as an agonist for E-selectin expression, while tetra-acylated mono-phosphoryl structures LPS_1_,_435/1_,_449_ act as antagonists [47]. Interestingly, structurally similar, penta-acylated, mono-phosphorylated LPS forms from *P. gingivalis* and the genus *Bacteroides* (from the gastrointestinal tract) caused vastly different types of innate immune reactions that were ascribed to subtle differences in the molecular weight fatty acid content of A-LPS [48]. This supports further the view that *P. gingivalis* A-LPS is unlikely to cause sepsis and formation of intra-abdominal abscesses in the host.

The different isoforms of LPS can also affect periodontal pathogenesis by disrupting pattern recognition receptors (PRRs) such as LPS-binding protein (LBP) [49,50]. LPS_1_,_4__35/1_,_449_ down regulated recombinant human (rh) LBP-induced IL-6 and IL-8 mRNAs significantly more than *P. gingivalis* LPS_1_,_690_ [50]. *P. gingivalis* LPS_1_,_690_-rh LBP interaction also caused a dramatic up regulation of the transcript of the cell surface molecule CD180 and a significant down regulation of the myeloid differentiation factor 1 (MD-1) transcript. This probably occurred through fine-tuning of the CD180–MD1 complex and appropriate TLRs [50]. LPS_1_,_690_ was shown to stimulate LBP in HOKs by affecting the signalling pathways of NF-ĸB and p38 MAPK whereas LPS_1_,_435/1_,_449_ was unable to perform this task [51]. Changing the lipid A structure of *P. gingivalis* may, therefore, be a crucial strategy for this keystone periodontal pathogen to escape from the hostile innate host defences.

Interestingly, an oral infection in apoliporotein E knockout (ApoE^−/−^) mice with a *P. gingivalis* strain expressing antagonistic lipid A caused vascular inflammation, macrophage accumulation, and progression of atherosclerosis [52], whereas a strain producing agonistic lipid A increased the levels of pro-inflammatory mediators and activated the inflammasome in a caspase-11-dependent manner. This led to host cell lysis and reduced bacterial survival. Thus, *P. gingivalis* avoided immune detection and promoted chronic inflammation in the vasculature. Chronic diseases may, therefore, be ascribed to pathogen-related strategies for immune evasion leading to low-grade inflammation.

## Differences in lipid A have different effects on TLR signalling

The innate immune system senses the invasion of pathogenic microorganisms via TLRs that recognize specific PAMPs. Heterogeneity in the *P. gingivalis* A-LPS can determine the virulence of this bacterium. This is seen especially via the elegant experiments related to hemin bioavailability [25]. Thus, *P. gingivalis* LPS_1_,_435/1_,_469_ and LPS_1_,_690_ can modulate TLR2 and/or TLR4 depending on the immediate environmental conditions and the cell type [25,30,47,50] to facilitate (epithelial/matrix) barrier breakdown for its access to its eventual periodontal niche. Tetra- and penta-acylated lipid A structures of *P. gingivalis* differentially activated TLR4-mediated NF-kB signal transduction and modulated the expression of IL-6 and IL-8 in HGFs [4]. TLR2 and cluster of differentiation (CD) 14 mRNA were regulated differentially in human gingival epithelia while the modulation of hBD-2 expression was suggested to occur through co-operation of both TLR2 and TLR4 [46]. In another study, multiple lipid A species interacted functionally with both TLR2 and TLR4 [15]. However, as already mentioned, the TLR2 agonist activity in *P. gingivalis* is most likely due to a lipoprotein, and treatment of LPS with lipoproteinase as free LPS substantially attenuated the TLR2 engaging activity [17]. These findings explain previous observations that *P. gingivalis* LPS can act both as a TLR4 agonist and antagonist, with an end result of varying cytokine secretion profiles [5,11,12]. Also in HOKs, *P. gingivalis* LPS_1_,_690_-induced LBP expression occurred through both TLR2 and TLR4 [51], while recombinant rh LBP significantly up regulated the expression of IL-6 and IL-8 in HOKs through the TLR2 signalling pathway [50]. These observations repeatedly highlight the adaptability of *P. gingivalis* not only to disrupt cell-specific barriers but also to select the level of potency conducive to the required inflammophillic milieu and disease initiation/progression. The shape of the lipid A component of LPS has been proposed to determine the physiological functioning of LPS. Conical-shaped LPS (e.g. from *E. coli*) may stimulate cells through TLR4 while cylindrically shaped LPS e.g. from *P. gingivalis* may stimulate cytokine production through TLR2 [53]. Strictly speaking, cylindrical LPS molecules, albeit from other bacterial sources (*Rhodobacter sphaerodides*), demonstrate antagonistic properties at TLR levels. Accordingly, the LPS from *P. gingivalis* can act as a TLR2 or TLR4 agonist or antagonist depending upon the TLR cell type being activated in the host, or if multiple bacterial lipid A species are present. The joint signalling pathways elicited by TLR2 and TLR4 agonists may diverge to enable distinct patterns of expression, a property elicited by *P. gingivalis* A-LPS heterogeneity.

## LPS influences host cell autophagy processes

*P. gingivalis* often subverts autophagy processes in host cells in order to survive. In a study by Blasi et al. [54], variants of *P. gingivalis* LPS altered lipidation of autophagy protein, i.e. microtubule-associated protein 1 light chain 3 (LC3). Whereas LPS_1_,_690_ stimulated production of very large autophagic protein LC3-positive vacuoles and cargo sorting protein MREG puncta in macrophages, the LPS_1_,_690_-mediated LC3 lipidation was reduced when LPS_1_,_435/1_,_449_ was present. This indicated that the prevalence of a particular LPS moiety could affect the degradative capacity of host cells, thereby influencing bacterial survival. This is noteworthy since peripheral blood mononuclear cells from periodontitis patients show increased levels of autophagy-related gene expression and high levels of mitochondrial reactive oxygen species, with further increase in LC3 upon stimulation of gingival fibroblasts with *P. gingivalis* LPS [55]. The ability to dysregulate autophagy in phagocytic cells has implications for development of distant organ inflammatory diseases in which either *P. gingivalis* or its LPS have been detected or been tested experimentally [56,57].

## A-LPS provides serum resistance to *P. gingivalis*

*P. gingivalis* is unique in that it displays commonality for the biosynthesis of A-LPS and the glycosylation of Arg gingipains [58]. Given that these two virulence factors, independently of each other, can be potent immune effectors in the host, why has *P. gingivalis* acquired this co-operation with its LPS and Arg gingipains as well? Experiments conducted with deletion mutants in the loci *porR* (PG1138) and *wbpB* (PG2119), which are devoid of A-LPS but possess O-LPS [59,60], exerted less gingipain enzymatic activity. These loci are, therefore, probably involved in the biosynthesis of A-LPS [60,61]. Furthermore, the mutants were susceptible to killing by the complement system, suggesting that A-LPS has a role in serum resistance to *P. gingivalis* [61,62]. Also, in the non-pigmented *P. gingivalis* strain HG66, which lacks A-LPS but possesses O-LPS, a nonsense mutation in the *wbpB* gene was detected, and Wbp pathway gene mutants were found to be A-LPS deficient [60]. Both penta-acylated lipid A and non-phosphorylated lipid A failed to activate TLR4 and provided the pronounced ability of these bacteria to resist polymorphonuclear neutrophil-mediated killing. The above experimental outcome suggests that A-LPS and Arg gingipains may be acting as switches that exert influence on the endotoxin/exotoxin activity for maintaining bacterial survival and retaining pathogenicity. The pigment of this bacterium plays a vital role in maintaining A-LPS heterogeneity and therefore its potent virulence.

## A-LPS affects production of OMVs

The main component of the outer leaflet of outer membrane vesicles (OMVs) is LPS, while the inner leaflet consists of a layer of phospholipids. Rangarajan et al. [27] suggested a novel mechanism for the production of OMVs in *P. gingivalis* implying dephosphorylation of the lipid A region of A-LPS that is controlled or regulated by the OM protein PG0027. This may be responsible for destabilization of the outer membrane followed by blebbing and generation of OMVs. Such an explanation is based on PG0027, which seems to control the amount of phosphorylated and non-phosphorylated species of lipid A in A-LPS, and thereby the production of OMVs in *P. gingivalis*. It is also plausible that these vesicles are produced in regions of the outer membrane enriched in A-LPS that are devoid of non-phosphorylated lipid A. Conversely, dephosphorylation of lipid A through a PG0027-dependent process may be required for optimal processing of OMVs. The relative proportions of non-phosphorylated and phosphorylated lipid A are obviously crucial for outer membrane blebbing and generation in *P. gingivalis.*

## Biosynthesis of A-LPS and glycosylation of Arg gingipains share common steps

Gingipains are important virulence factors of *P. gingivalis* in addition to LPS. Genesis of these factors may share common pathways as suggested previously, in respect to µ-oxo-bisheme, responsible for the black pigment. Since a monoclonal antibody (MAb1B5) raised against the Arg-gingipain RgpA cross-reacted with a cell-surface polysaccharide of *P. gingivalis* strain W50 (A-LPS) [24,63], it was suggested that the maturation pathway of the Arg-gingipains could be linked to the biosynthesis of a surface carbohydrate [24]. The cross-reacting APS, different from the LPS and serotype capsule polysaccharide, was found to be a phosphorylated branch of mannan backbone. The backbone consisted of α-1,6-linked mannose residues, and the side chains of α-1,2-linked mannose oligosaccharides of various lengths. One of the side chains of the repeating unit carried Manα1–2Manα1-phosphate linked through phosphorous to a mannose backbone at position 2. This lead to the proposal that the Manα1–2Manα1-phosphate fragment constitutes part of the epitope recognized by MAb1B5. This phosphorylated branched mannan represented a new polysaccharide that appears immunologically connected to the post-translational additions of Arg-gingipains [24,63]. Accordingly, there seem to be common steps in the biosynthesis of A-LPS and the glycosylation of Arg gingipains in *P. gingivalis*.

## Dysregulation of the TLR function may cause adverse pregnancy outcomes

*P. gingivalis* has been associated with adverse pregnancy outcomes (APOs) [64,65]. A hemin-rich medium promotes the growth of TLR4 antagonist forms of *P. gingivalis* [25]. This may have consequences for the placenta, which is a blood-rich tissue with abundance of red blood cells. The placental environment may, therefore, be rich in tetra- and penta-acylated residues in the A-LPS of *P. gingivalis* [64]. The ability of *P. gingivalis* to generate two different isoforms of A-LPS that either activate or antagonize TLR4 could be particularly important to the maternal–foetal interface [64]. *P. gingivalis* dysregulation of the TLR function in uterus, placenta, and foetal membranes may promote APO [64–67]. In rats, LPS from *P. gingivalis* increased maternal blood pressure, induced placental and foetal growth restriction, and increased foetal resorptions, without inducing proteinuria and inflammation [68]. It is likely, therefore, that *P. gingivalis* LPS and/or A-LPS may play a role in pregnancy complications related to periodontal pathogens.

## Concluding remarks

A-LPS of *P. gingivalis* is remarkably heterogeneous. This was originally discovered following various biochemical approaches used to extract LPS. The different isoforms of LPS varied biochemically because of the methodology utilized, and the environmental factors experienced during their culture, for example presence of hemin and temperature conditions. Analytical techniques were instrumental in deducing that *P. gingivalis* lipid A consists of multiple structures including a phosphorylated penta-acylated form and a phosphorylated tetra-acylated form. These species were absent in O-LPS. The fact that different strains of *P. gingivalis* displayed varying capacity to elicit host inflammatory responses also suggests that the structure of the major virulence factors O-LPS and A-LPS could differ. This property is reflected in their interactions with the PRRs. The variation could actually be one of multiple strategies that *P. gingivalis* uses to evade innate host defence in periodontal tissues, thereby contributing to periodontal pathogenesis.

The recognition that there are at least two different forms of *P. gingivalis* LPS, O-LPS and A-LPS, is significant. This is particularly important because A-LPS has been shown to have important effects on virulence through its effect on OMV production, serum resistance, and by serving as a matrix for deposition of µ-oxo-bisheme on the cell surface. The heterogeneous lipid A structures may also have distinct and opposing effects on TLR receptors playing a critical role in the early innate immune response to invading pathogens. Only subtle changes in lipid A structures are required to influence the host immune response to a large extent, and it has been speculated that *P. gingivalis* uses the ability of changing its lipid A composition to paralyze local pro-inflammatory cytokine production, thereby gaining access to periodontal tissue. Although the mechanisms that control the natural lipid A heterogeneity in *P. gingivalis* are not clear, they may exert a form of immunomodulation that helps *P. gingivalis* adapt and survive in different host environments and induce chronic inflammation.

Unfortunately, there is no evidence yet to demonstrate that a shift in lipid A classes occurs in subgingival plaque samples when comparing healthy/gingivitis or chronic periodontitis sites. This is a critical biological correlate required to be demonstrated before arguments about the importance of different lipid A classes can be accepted. Lipid A preparation from wild-type *P. gingivalis* contains unphosphorylated and phosphorylated lipid A constituents as well as tetra- and penta-acylated classes. Considering the documented opposing effects of these preparations, and the likelihood of the presence of multiple species within each isolate, it is not clear how the combinations of lipid A classes affect the engagement of innate immune receptors in its primary niche and/or at disparate sites. One possibility is certain, *P. gingivalis* is a ‘microbial wizard’ at adapting to tissue-specific cell receptor signal transduction pathways to colonise niches where growth conditions may be less than adequate albeit *in vitro, in vivo* animals, or the human host. This wizard’s wand may well be the heretogenity displayed in *P. gingivalis* lipid A structures.
